# Oral health related behaviors among adult Tanzanians: a national pathfinder survey

**DOI:** 10.1186/1472-6831-9-22

**Published:** 2009-09-14

**Authors:** Joyce R Masalu, Emil N Kikwilu, Febronia K Kahabuka, Ahadieli R Senkoro, Irene A Kida

**Affiliations:** 1 School of Dentistry, Muhimbili University of Health and Allied Sciences, P.O. Box 65014, Dar es Salaam, Tanzania; 2 Central Oral Health Unit; Ministry of Health, United Republic of Tanzania, P.O. Box 273, Dar es Salaam, Tanzania

## Abstract

**Background:**

The oral health education programs which have been organised and delivered in Tanzania were not based on a thorough understanding of behaviours which influence oral health. Therefore, evaluation of these programs became difficult. This study aimed at investigating the oral health related behaviours and their determinants among Tanzanian adults.

**Methods:**

A national pathfinder cross sectional survey was conducted in 2006 involving 1759 respondents from the six geographic zones of mainland Tanzania. Frequency distributions, Chi square and multiple logistic regression analyses were performed using SPSS version 13.0.

**Results:**

The rates of abstinence from alcohol for the past 30 days and life time smoking were 61.6% and 16.7% respectively, with males being more likely to smoke (OR 9.2, CI 6.3 -12.9, p < 0.001) and drink alcohol (OR 1.5, CI 1.2 -1.8, p < 0.001). Multiple regression analysis revealed that; having dental pain (OR 0.7, CI 0.5-0.8; p < 0.001) and being minimally educated (OR 0.48, CI 0.4-0.6; p < 0.001) reduced the likelihood of having a high sugar score. Whereas being male (OR 1.5, CI 1.2- 1.8; p < 0.001), urban (OR 1.9, CI 1.5 -2.3; p < 0.001), and young (OR 1.5, CI 1.2 -1.8; p < 0.001) increased the likelihood of having a high sugar score. Urban residents were less likely to take alcohol (OR 0.7, CI 0.6-0.9; p < 0.01), or smoke cigarette (OR = 0.7, CI = 0.6-0.9); less likely to be those who do not use fruits (OR 0.3, CI 0.2-0.4; p < 0.001); dental clinic (OR 0.5, CI 0.4-0.7; p < 0.001); factory made tooth brushes (OR 0.1, CI 0.08-0.17; p < 0.001) and toothpaste (OR 0.1, CI 0.1-0.2; p < 0.001) than their rural counterparts. More rural (13.2%) than urban (4.6%) residents used charcoal.

**Conclusion:**

The findings of this study demonstrated social demographic disparities in relation to oral health related behaviors, while dental pain was associated with low consumption of sugar and high likelihood to take alcohol.

## Background

Since early 1980s, the Ministry of Health in Tanzania has been advocating the provision of oral health education to all Tanzanians. Most of the oral health education programs which have been organised and delivered in Tanzania were not based on a thorough understanding of behaviours conducive or detrimental to oral health among Tanzanian population. Rather they were based on the studies on behaviours and disease conditions which were limited to groups of segments of the population. Consequently, they have not been systematically evaluated for their effectiveness. To improve on the organization and delivery of oral health education, a national pathfinder survey was organised in order to gather baseline information that will guide future planning of oral health services. This study explores oral health related behaviours as part of the national pathfinder survey.

Oral diseases are linked with the lifestyle of an individual person, and their prevention depends on adopting lifestyles that are conducive to oral health. The important oral health behaviours that have been shown to have a positive impact on oral health include tooth-brushing with fluoridated toothpaste, inter-dental cleaning by tooth picks or dental floss, and dental attendances [1-4]. Consumption of fresh fruit is important for the maintenance of good health, moreover being less cariogenic; fruits may be good alternatives of sugared snacks for the prevention of dental caries [5]. Much is known about the oral damaging effects of alcohol & tobacco consumption [6]. Excessive consumption of alcohol, especially hard drinks and tobacco has been strongly linked with occurrence of oral cancer. Other detrimental practices such as use of charcoal abrasives are still prevalent in some societies and their prolonged use may have a negative impact on oral health. A variety of factors account for individual differences in the propensity to undertake oral health behaviours, including demographic, social, emotional and personality related factors as well as cognitive factors [7]. Socio-demographic variables have been shown to have associations with the performance of oral health related behaviours in the adolescent and young adult populations. In industrialized countries, females and those who report higher levels of education and income are more likely to engage in oral hygiene behaviours, less smoking and less frequent sugar consumption than males and people of lower socio-economic status [8,9]. In developing countries however, intake of sugared snacks is generally most common among females, the higher educated and among those residing in urban areas [10,11]. Understanding the influence of demographic factors on peoples' behaviour is crucial in planning intervention programs. This information guides the targeting of health education messages to those who have a high likelihood of performing unhealthy behaviours across demographic factors.

Studies conducted in sub-Saharan African countries have shown that low to moderate proportions of the populations confirm daily intake of sugared foods and drinks [10,12]. Much is desired to counter the cariogenic effects of sugars on teeth by using Fluorides. Moreover it is a well established fact that Fluoride tooth paste has accounted for much of the decline in caries experience in many industrialised countries. In addition, fluoride toothpaste can be distributed to many retailer shops together with other domestic consumables. However, affordability of the toothpaste and availability of the fluoride ion in toothpaste in its bioactive form pause a challenge to many low income countries [13]. In this era of trade liberalization [14], few will disagree that caries experience is likely to increase in low income countries that have access to sugars and minimal exposure to fluorides. Authorities in Tanzania have to strike a balance between opening links with global markets and building healthy public policies that protect oral health. These policies should promote the use and availability of affordable fluoride tooth paste. In addition, they should support efforts aimed at raising the awareness of communities on aetiology and prevention of dental caries in order to improve the rational use of sugar containing foodstuffs and fluoride containing toothpaste.

Studies conducted in Africa indicate that majority and more females than males [15] engage in daily tooth cleaning; with more rural than urban residents using chewing sticks [16]. This indicates residents in the continent of Africa could benefit from routine use of fluoridated toothpaste for dental caries prevention. In early 1990's the lifetime prevalence of dental attendance among adult Tanzanian population was 51% and 43% among men and women respectively [17]. Majority seek dental care for pain relief when the carious lesion has destroyed the tooth crown, and often with abscess formation. In most cases, the treatment rendered is tooth extraction [18]. Therefore health educators need to emphasize on the importance of dental visits for routine check-up to allow early detection and treatment of dental caries by preventive methods such as fissure sealants and restoration.

Regarding life time tobacco use, prevalence across studies in some African countries vary from levels below 10% to over 30% with higher rates observed among males than females and among urban than rural residents [11,19,20]. A common assumption all over sub-Saharan Africa is that cigarette smoking is the habit of affluent people, whereas other forms of tobacco such as snuff tobacco are most common among the less educated and those residing in rural areas. However, these consumption patterns are likely to change with time due to shift of market targets. As correctly put by Legressley et al 2008, the marked decline in the sales of tobacco in the industrialised world seems to have been compensated by a rapid development of new generation of smokers in Sub-Saharan Africa [21].

The link between behaviour and oral health can not be over-emphasized. The task ahead of us is to understand behaviour and its potential determinants that would be targeted by oral health interventions. The government of Tanzania in its Policy guidelines for oral health indicated its intention to intensify oral health education activities in the country [22]. In line with this desire, understanding the behaviours affecting oral health status will be core to the structuring of evidence based oral health education programmes. The aim of this study therefore was to investigate oral health related behaviours and their determinants among Tanzanian adults.

## Methods

### Selection of study sites and Sampling procedure

The national pathfinder survey methodology described in the WHO Oral Health Surveys - Basic Methods [23] was used to select sampling sites and sample size. However the number of rural clusters was slightly increased to redress the urban rural disproportion. Tanzania mainland has 6 geographical zones. From two zones, 2 study sites, Dar es Salaam and Mbeya cities were purposively selected. Six clusters, 4 from Dar es Salaam and 2 from Mbeya cities were purposively selected to represent urban population. From each of the remaining four zones, one region was randomly selected, as a study site from a list of the regions constituting a given zone. From each of the study site, 2 villages were purposively selected as clusters for rural population. All adults in the selected clusters were eligible for inclusion in the study. A total of 14 clusters, 6 from urban and 8 from rural areas were selected. From each cluster, 150 adults aged 18 years and above, were to be interviewed. Therefore, a total of 2,100 subjects (900 urban, 1200 rural) were targeted.

To facilitate stratification of respondents by sex and age, each interviewer was provided with a matrix table for sex: (*male and female)*; and 5 age-groups: (*18-25, 26-35, 36-45, 46-55, and 56+)*. Each age and sex category had a predetermined number of 15 respondents. The interviewer had to tally in the appropriate age and sex category to which an interviewee belonged. At the end of the study period, some age and sex categories in the matrix were not completely filled due to difficulties of getting people in their households during the day time. Only 1,759 out of the targeted 2,100 adults were interviewed, giving a response rate of 84%.

### Procedure used to select study participants

This was a house-to-house survey. Cities, towns and villages in Tanzania are divided into smaller administrative units of 10-20 households called streets. Interviewers reported to the city, town or village authorities who assigned one street leader to lead the interviewers from house to house under his/her street until all the adults in a street who were present at the time of the study were interviewed. The street leader then handled over the responsibility of leading the interviewers to the next street leader. This process continued until the interviewers had interviewed the required number of adults of each age-group and sex category.

### Ethical clearance and procedure for obtaining informed consent from respondents

The ethical clearance for conducting this study was obtained from the Ministry of Health of the United Republic of Tanzania. The street leaders who led the interviewers introduced them to the family members and interviewers explained the aim of the visit. After the household members had understood the aim of the study, all members aged 18 years and above were requested to participate in the study by responding to the questions posed by interviewers. Members were informed that they were free to participate/not participate. It was agreed before commencement of the study that a person who would accept to be interviewed after explanations would have consented to participate in the study.

### Development of a questionnaire

The data reported in this study is part of a national pathfinder survey. The items measuring oral related behavior and dental pain experience were adopted from the WHO simplified oral health questionnaire for adults [24] and amended for use in Tanzania. Included also in this questionnaire were demographic variables.

The English questionnaire was translated to the national language and pre-tested among 20 adults in each of the 6 administrative zones for meaning and clarity. Pre-testing was conducted in all the 6 zones to capture possible differences in the interpretation of words and phrases. The final version was administered twice to a group of respondents for reliability in terms of temporal stability with kappa values ranging from 0.80-1.0 indicating high temporal stability of the instrument.

### Data analysis

#### Construction of dummy variables and coding for analysis

The questionnaire assessed the consumption of sugary snacks, fruits, tobacco and alcohol, dental visit, use of tooth brush, mswaki (chewing sticks), toothpaste, charcoal, tooth picks and dental floss. Consumption of sugared snacks & drinks, and fruits was measured on a scale ranging from (1) = rare/never to (6) = several times a day. Tobacco and alcohol consumption was measured from (1) = never to (6) = every day. To allow for cross-tabulation and logistic regression the ordinal scale was dichotomized to "category 0" including the original score (1), and "category 1" including the original scores (2-6). Dichotomized score for fruit consumption were then reversed (yes = 0, and No = 1). Dental visit, use of factory made tooth brush, chewing stick, dental floss, toothpicks, tooth paste and charcoal were measured on a dichotomized scale; (0) = yes and (1) = No. There were six items assessing the consumption of sugared snacks and drinks (Biscuits/cakes, doughnuts, sodas, jam/honey, chewing gums and sweets/chocolate; [Table 1]. The six items were added up to construct a sugar score (mean 14.5; SD 5.5). The score ranged from 6 = rare or no consumption to 36 indicating frequent consumption of all the six food items. The score was dichotomized at approximately median split, into (0) = rare including score 6-13 and (1) = frequent consumption of sugar including score 14 - 36.

**Table 1:** Frequency distribution of participants by experience of dental pain and socio-demographic characteristics (n = 1759)

**Pain experience and socio-demographic characteristics**	**n**	**%**
**Experience of pain**		
• Never had dental pain	725	41.2
• Had dental pain	1034	58.8
**Socio-demographic characteristics**		
• 37 yrs and above	864	49.1
• 18-36 years	895	50.9
• Rural	1027	58.4
• Urban	732	41.6
• Female	896	50.9
• Male	863	49.1
• Secondary education and above	293	16.7
• Primary education or less	1466	83.3

The independent variables considered in this study were residence, sex, age, education and experience of dental pain/discomfort for the past 12 months. These were coded as follows: Residence (0) = rural, (1) = urban; Sex (0) = female, (1) = male; age was dichotomized at median split into young adults (18-36 years) and older adults (37+ years), then coded as (0) = older adults (1) = young adults, dental pain experience was dichotomized into (0) = no pain and (1) = yes pain experienced. Education was categorized as (0) = secondary education or more, and (1) primary education or less.

### Statistical analysis

Data were analyzed using Statistical Package for Social Sciences version 13. Cross-tabulation and chi-square statistics were used to assess bivariate relationships. Multivariate analysis was conducted using multiple logistic regressions. The p-value for statistical significance was set at 0.05. Forced entry multiple logistic regression (enter method) analyses were performed with 95% confidence intervals (CIs) given for Odds Ratios (ORs) indicating a possibility of an effect if both values are either greater or less than 1. To check for the effect of other independent variables on the dependent variable both unadjusted and adjusted ORs and their corresponding CIs were computed (only the adjusted ORs were reported because there were no much differences between the two). To estimate the likelihood of an individual carrying a risk to get a bad outcome, the worst scenario of an independent variable was coded (1) and the bad outcome of the dependent variable was also coded (1), while maintaining (0) as the first and reference category in regression analysis.

## Results

The distribution of participants by age and sex in urban and rural areas was determined by pre- stratification into allowable quotas with minor variations in each cluster and strata. Distribution of participant's socio-demographic variables and experience of dental pain is shown in Table 1. Frequency distributions of participants according to alcohol consumption showed that 61.6% of all participants did not have an alcoholic drink for the past 30 days prior to the interview. Among those who reported to have taken alcohol in the past 30 days the rates of consumption per day were 5 or more, 4, 3, 2, and 1 drinks (among 10.2%, 4%, 6.5%, 8.9% and 8.7% respectively). With the exception of cigarette that was reported to ever been consumed by 16.7% of the respondents, other forms of tobacco namely cigars, pipe, snuff and chewing tobacco were consumed by a small proportion of individuals. Frequency distribution of participants' consumption of sugared snacks and fruit is displayed in Table 2.

**Table 2:** Distribution of respondents by frequency of consumption of sugary drinks/foods, number of respondents and percentage in parenthesis

	**Very rarely or never** **n (%)**	**Few times a month** **n (%)**	**Once a week** **n (%)**	**Several times** **a week** **n (%)**	**Everyday** **n (%)**	**Very frequently** **n (%)**
Fruit	109 (6.2)	352 (20.0)	211 (12.0)	578 (32.9)	330 (18.8)	179 (10.2)
Biscuit/cake,	785 (44.6)	373 (21.2)	201 (11.4)	262 (14.9)	86 (4.9)	52 (3.0)
Doughnut	356 (20.2)	228 (13.0)	154 (8.8)	356 (20.2)	497 (28.3	168 (9.6)
Soft drinks	449 (25.5)	383(21.8)	229 (13.0)	345 (19.6)	229 (13.0)	124 (7.0)
Chewing gums	961 (54.6)	291 (16.5)	120 (6.8)	188 (10.7)	118 (6.7)	81 (4.6)
Sweets/chocolate	855 (48.6)	327 (18.6)	134 (7.6)	232 (13.2)	119 (6.8)	92 (5.2)
Jam or honey	1366 (77.7)	232 (13.2)	51 (2.9)	49 (2.8)	36 (2.0)	25 (1.4)

Cross tabulation of oral health related behaviors by residence and sex is displayed in Table 3 &4. Participants reported to consume fruits at least once a week (88% and 63.7%) for urban and rural respectively, with no significant differences between sexes. Biscuits/cakes were reported to be consumed by 39.9% among urban and 30.1% of rural residents, with more males than females in rural areas consuming these products (p < 0.05). Seventy four percent and 61.6% of urban and rural residents respectively consumed doughnuts with more males than females in rural areas taking them (p < 0.05). More urban (74.6%) than rural (37%) residents consumed soft drinks, with more rural males than females more likely to consume the products. Honey and jam was consumed by small proportions of urban and rural residents. Alcohol consumed in the past 30 days was reported by 33.6% of urban and 41.9% of rural residents with urban males more likely to drink than females (p < 0.001). Fourteen percent of urban and 18.4% of rural residents ever smoked cigarettes, in both urban and rural areas significantly (p < 0.001) more males than females ever smoked. Mswaki was reported to be used by 15.7% by urban and 49.6% of rural residents with more rural females than males more likely to brush by using mswaki (p < 0.001). Nearly 95%, of urban and 66.4% rural residents reported to use factory made tooth brushes with no significant differences between sexes in both settings. Toothpaste was reported to be used by 94.1% of urban and 66.4% of rural residents with no sex difference across localities of residence. The prevalence of charcoal use was 4.6% and 13.2% among urban and rural residents respectively with rural females than males more likely to brush their teeth using charcoal. About half (49.6%) of urban and 36.8% of rural residents ever visited a dental clinic with more urban females than males more likely to visit the clinic (p < 0.05). Behaviors that were found to be performed by a sizable proportion of participants were subjected to regression analysis as outcome variables.

**Table 3:** Distribution of urban and rural residents according to consumption of sugared foods and fruits at least once a week by sex

	**Urban (n = 732)**			**Rural (n = 1027)**		
	
**Oral health related behavior**	**Female** **n (%)**	**Male** **n (%)**	**Total** **n (%)**	**Female** **n (%)**	**Male** **n (%)**	**Total** **n (%)**
Fruit	321(88.7)	323 (88.3)	644 (88.0)	327(61.2)	327 (66.3)	654 (63.7)
Biscuit/cake	150 (41.4)	142 (38.4)	292 (39.9)	138 (25.8)*	171 (34.7)	309 (30.1)
Doughnut	268(74.0)	272 (74.1)	542 (74.0)	313(58.6)*	320 (64.9)	633 (61.6)
Soft drink	260 (71.8)	286 (77.3)	546 (74.6)	136 (25.5)**	245 (49.7)	381 (37.1)
Jam honey	24 (6.6)	21 (5.7)	45 (6.1)	6 (1.1)*	10 (2.0)	16 (1.6)
Chewing gum	102 (28.2)	95 (25.7)	197 (26.9)	122 (22.8)**	188 (38.1)	310 (30.2)
Chocolate/sweets	94 (26.0)*	71 (19.2)	165 (22.5)	193 (36.1)*	219 (44.4)	412 (40.1)

Chi square: * p ≤ 0.05, ** p ≤ 0.001

**Table 4:** Distribution of urban and rural residents according to consumption of alcohol, cigarettes and oral hygiene practices by sex

	**Urban (n = 732)**			**Rural = 1027**		
	
**Oral health related behavior**	**Female** **n (%)**	**Male** **n (%)**	**Total** **n (%)**	**Male** **n (%)**	**Female** **n (%)**	**Total** **n (%)**
Alcohol in 30 days	90 (24.9)**	156 (42.2)	246 (33.6)	213 (43.2)	217 (40.6)	430 (41.9)
Ever used Cigarette	9 (2.5)**	95 (25.7)	104 (14.2)	157 (31.8)	32 (6.0)**	189(18.4)
Mswaki#	59 (16.3)	56 (15.1)	115 (15.7)	215 (43.3)	294 (55.1)**	509 (49.6)
Tooth paste	341 (94.2)	348 (94.1)	689 (94.1)	325 (65.9)	357(66.9)	682 (66.4)
Charcoal	16 (4.4)	18 (4.9)	34 (4.6)	47 (9.5)	89 (16.7)*	136(13.2)
Toothpick	139 (38.4)*	99 (26.8)	238 (32.5)	96 (19.5)	56 (10.5)**	152 (14.8)
Dental visit	195 (53.9)*	168 (45.4)	363 (49.6)	173 (35.1)	205 (38.4)	378 (36.8)
Factory toothbrush	341 (94.2)	354 (95.7)	695 (94.9)	340 (69.0%)	342 (64.0)	682 (66.4)

Chi square: * p ≤ 0.05, ** p ≤ 0.001, #^.... ^plant twig used as a tooth brush

Multiple regression analysis controlling for age, sex, residence, education level and experience of dental pain or discomfort showed significant effects in terms of Adjusted Odds Ratios (OR) and 95% CIs of most independent variables on the oral health related behaviors shown in Table 5 &6. Corresponding Nagelkerke R^2 ^for each model are also displayed in Table 5 &6.

**Table 5:** Adjusted Odds Ratios (OR) and 95% Confidence intervals (CI) for participants' intake of Biscuits, doughnuts, soda, chewing gums, chocolate and sugar score according to socio demographic characteristics and pain experience

	**Biscuits/cakes**	**Doughnuts**	**Soda**	**Chewing gum**	**Chocolate**	**Sugar score**
	**OR (95%CI)**	**OR (95%CI)**	**OR (95%CI)**	**OR (95%CI)**	**OR (95%CI)**	**OR (95%CI)**

Dental pain	0.8 (0.6-0.9) **	0.8 (0.6-0.9) **	0.8 (0.6-0.9) *	0.6 (0.5-8.0) **	0.9 (0.7-1.1)^*ns*^	0.7 (0.5-0.8)**
Residence	1.5 (1.2-1.8) **	1.7 (1.4-2.1) **	4.9 (3.9-6.6) **	0.8 (0.6-0.9) *	0.4 (0.3-0.5) **	1.9 (1.5-2.3)**
Age	2.2 (1.8-2.7) **	1.5 (1.2-1.8)^*ns*^	2.0 (1.7-2.5) **	2.8 (2.2-3.5) **	2.9 (2.4-3.7) **	2.7 (2.2-3.4)**
Sex	1.2 (0.9-1.5)^*ns*^	1.2 (0.9-1.4)^*ns*^	2.2 (1.8-2.7) **	1.5 (1.2-1.9) **	1.1 (0.9-1.4)^*ns*^	1.5 (1.2-1.8)**
Education	0.9 (0.7-1.1)^*ns*^	0.9 (0.6-1.1)^*ns*^	0.6 (0.4-0.8) *	0. 8 (0.6-1.1)^*ns*^	0.8 (0.6-1.1)^*ns*^	0.5 (0.4-0.6)**
Nagelkerke *R*^2^	0.07	0.047	0.256	0.103	0.128	0.165

Multiple logistic regression analyses: * p ≤ 0.05, ** p ≤ 0.001, ns = Not statically significant

**Table 6:** Adjusted Odds Ratios (OR) and 95% Confidence intervals (CI) for participants intake of Tobacco, alcohol, dental visit, tooth-brush, toothpaste and fruit use according to socio demographic characteristics and pain experience

	**Tobacco**	**Dental visit**	**Alcohol**	**Toothbrush**	**Tooth-paste**	**Fruit**
	**OR (95%CI)**	**OR (95%CI)**	**OR (95%CI)**	**OR (95%CI)**	**OR (95%CI)**	**OR (95%CI)**

Dental pain	1.3 (0.9-1.7)^*ns*^	0.1 (0.1-0.2) **	1.3 (1.1-1.6)*	0.7 (0.5- 0.9) *	0.9 (0.7-1.2)^*ns*^	0.9 (0.7-1.1)^*ns*^
Residence	0.7 (0.6-0.9) *	0.5 (0.4-0.7) **	0.7 (0.6-0.9) **	0.1 (0.1-0.2) **	0.1 (0.1-0.2) **	0.3 (0.2-0.4) **
Age	0.6 (0.5-0.8) **	2.0 (1.6-2.5) **	0.7 (0.6-0.8) **	0.3 (0.2-0.4) **	0.4(0.3-0.5) **	0.5 (0.4-0.6) **
Sex	9.2 (6.3-12.9) **	1.2 (0.9-1.5)^*ns*^	1.5(1.2-1.8) **	0.8 (0.6-0.9) *	1.1 (0.8-1.4)^*ns*^	0.9 (0.7-1.0)^*ns*^
Education	1.5 (0.9-2.2)^*ns*^	2.4 (1.7-3.2) **	1.2 (0.9-1.6)^*ns*^	5.0 (2.7-9.9) **	5.3 (2.8-9.9)**	2.6 (1.7-3.9) **
Nagelkerke *R*^2^	0.215	0.315	0.041	0.279	0.246	0.157

Multiple logistic regression analyses: * p ≤ 0.05, ** p ≤ 0.001, ns = Not statically significant

Dental pain significantly reduced the likelihood of consuming biscuits, doughnuts, soft drinks and chewing gums, likewise people with pain had a reduced likelihood of not attending dental treatment and more likely to drink alcohol [Table 5 &6].

Urban residents were significantly more likely to consume biscuits/cakes, doughnuts, and soft drinks, with highest odds ratios observed for soft drinks (OR 4.9, CI 3.9-6.6; p < 0.001). Urban residents were also less likely to take alcohol, or smoke cigarettes. They were less likely not to consume fruits, not to attend dental treatment and not to use factory made tooth brushes than their rural counterparts [Table 5 &6]

The young were more likely to use biscuits/cakes, doughnuts, soft drinks, chewing gums, chocolate, and visit dental clinic, but they were less likely to smoke cigarette and to take alcohol, with low likelihood of not using a factory made toothbrush or not eating fruits than old people [Table 5 &6].

Males were more likely to take soft drinks (OR 2.2, CI 1.8-2.7; p < 0.001), smoke cigarettes (OR 9.2, CI 6.3-12.9); p < 0.001), alcohol and chewing gums with less likelihood to be those who do not use a factory made toothbrush. The minimally educated individuals were less likely to consume soft drinks (OR 0.6, CI 0.4-0.8; p < 0.01) but were more unlikely; to eat fruits, visit a dental clinic, and unlikely to be none smokers. Moreover; they were more likely to be those who do not use a factory made tooth brush [Table 5 &6].

The summative index for sugar score also varied with the background variables in that; having dental pain and being minimally educated reduced the likelihood of having a high sugar score. Whereas being male, urban, and young increased the likelihood of having a high sugar score [Table 5]

## Discussion

This paper documents the associations between oral health related behaviors and socio-demographic factors among Tanzanian adults. The methodological strength of the present study includes the large sample size drawn from all the six geographical zones of mainland Tanzania. Using a WHO simplified oral health questionnaire for adults [24] makes the findings of this study comparable with those of other studies. A diverse range of oral health related behaviors studied offers substantial national baseline information for planning and scientific referencing. This study used the oral health surveys pathfinder methodology [23], which is scientifically less rigorous than the standard probability sampling methods. However; it is widely advocated by the World health organization especially when the information collected is for planning oral health services.

Lack of information about the non-respondents precludes any conclusion about a possible selection bias, although the response rate was high enough to assume that the target population is reflected with a reasonable degree of accuracy. The clusters were purposively selected to capture the diversity of characteristics, however the individuals were let to participate conveniently until the quota size was attained for each cluster. This could have introduced volunteer bias. Nevertheless the pre-stratification by age and sex in specified quotas might have redressed the bias to some extent. The present study relied on self reported information; a possibility of over and under reporting due to respondents' seeking social desirability could lead to bias. However temporal stability was checked with satisfactory reliability. The data might provide a reflection of oral health related behaviors among adult Tanzanians. However, as the respondents were drawn by non-probability sampling, the findings must be interpreted with caution when making direct generalizations to the whole country. Furthermore; at the point of analysis some ordinal and continuous variables were dichotomized to allow for logistic regression. This to some extent might have reduced the power and a better fitting of the data. The cut off points might have misclassified individuals to categories that they did not belong. Therefore the costs of dichotomization should not be ignored when interpreting these findings. Moreover most of the ORs were modest indicating that the differences between the categories were not very prominent. However the displayed differences could be useful in real life planning situations.

The findings of this study indicated that urban residents showed a high likelihood to snacking sugary foods and drinks, eat fruits, attend dental clinics, use factory made tooth brushes but were less likely to take alcohol or smoke cigarettes than their rural counterparts. The higher tendency of urban than rural residents to consume sugar was also reported in a study among Tanzanian University students [11], South Africans [25] and Ghanaian adolescents [10]. As correctly put by Holmboe-Ottesen [26], urbanization and globalization increase the consumption of sweet soda pops, biscuits and other snacks produced by multinational companies. In addition urban residents in developing countries are easily targeted by food adverts through the media hence become alternative consumers of confectionery that would otherwise not get an easy access to western markets [14]. Healthy public policies are necessary for monitoring the influx of sugary foods and drinks in Tanzania to protect consumers from irrational use of these commodities. Besides; reduction of sugar consumption fits into the common risk factor approach to disease prevention [27]. In this regard, reduction of sugar consumption will not only contribute to the prevention of dental caries but also other chronic lifestyle diseases. In another perspective, fear of high death tolls from the chronic conditions might reinforce the restriction of sugar intake and in so doing contribute to caries prevention.

Health promotion emphasizes the importance of supportive environments in enhancing people to choose healthier lifestyles. Therefore, health educationists have to consider the intricate mediating role of residence environment in shaping snacking behaviors. This study found that only a small proportion of individuals consumed sugary snacks and drinks very frequently. However with trade liberalization, this distribution might scale up to higher values especially in urban areas where the environment is conducive to promote the consumption of varieties of sugary snacks and drinks. Therefore deliberate efforts should be made to maintain these low levels of sugar consumption.

While it is recommended to eat fruits about five times a day [28], this study found 88% of urban and about 64% of rural residents consuming fruits at least once a week. Although fruits are known to be cultivated in rural areas, it was noted with concern that more urban than rural residents eat fruits. As also reported elsewhere [29], knowledge of the recommended frequency and perceived benefits of fruit intake might not be sufficient among the study participants and particularly rural residents. It is also important to note that unreliable transportation in rural areas leads to difficulties in moving goods from place to place. As a result; people depend largely on locally grown fruits of which their availability is seasonal. This disadvantage might have accounted for the low rates in fruit consumption among rural respondents.

Proportionately fewer rural as compared to urban residents used factory made toothbrushes and toothpaste. Alternatively; a higher proportion of rural residents used miswaki and charcoal than their urban counterparts. Rural residents in this study were also disadvantaged as regards utilization of dental services. As rural communities in many aspects represent less affluent societies, affordability and accessibility of dental services could be a challenge to the poor rural residents. Consequently, the immediate options tend to be self medication or hope that dental pain would disappear on its own [30]. Despite of a number of measures deliberated by the Ministry of health in its policy guidelines for oral health [22] studies conducted more than a decade ago on dental attendance rates in Tanzania portray a similar rural-urban disparity [17]. Left with constrained access to modern health facilities; rural residents also seek alternative medicine through traditional healers [31]. This rural-urban socio-economic gradient reflects among other things, a social inequality which puts rural residents at a disadvantage, whereby their opportunities are more or less confined to what can be locally available.

While other forms of tobacco were reported to be consumed by small fractions of the study sample, the prevalence of ever used cigarettes was 16.7%; which is almost similar to the rate reported in another study among Tanzanian university students [11]. This study also found males were more likely to smoke than females. However with the ever enduring multinational tobacco adverts; it will not be surprising in some years to come to have more smokers even among women. Smoking and alcoholism clustering reported by Myers et al [32], has also been found to be associated with rural residents in this study. Unfortunately, this adds up on the risks to the already disadvantaged society. Minimal recreation facilities in rural areas might have been compensated by smoking and alcoholism. Contrary to this line of thinking, Pootinger, [33] reported heavy drinking among sports club members. Exploring alcohol and tobacco information further this study also showed that dental pain increased the likelihood of drinking alcohol. Similar findings were also reported by Lahti [34]. Whether alcohol was used as a means to cub down the dental pain or rather the pain coexisted with other forms of misery which prompted the participants to drink, that is yet to be explored. However, it has been reported elsewhere that dental health detrimental behaviors correlate with the use of marijuana, smoking frequency, and engagement in antisocial behavior [35]. This clustering calls for a careful exploration of determinants of health behaviors. This information will help in structuring health promotion activities that will unearth what is rooted under the clusters of unhealthy behaviors. Although a higher proportion of educated people resided in urban areas and the minimally educated were more likely to smoke cigarettes, controlling for the potential confounders, this study also found that urban residents were less likely to be those who smoke, implying that being a rural dweller in itself added to the likelihood of smoking cigarettes. The whole scenario portrays a limited leeway for rural residents to live healthier lives. Viewing life in terms of its quality and fall into line with those believing in equity and equality in health; rural residents in Tanzania deserve a fresh look if they are to give a significant contribution to the achievement of the National Strategy for Growth and Reduction of Poverty.

The rural-urban disparity displayed by the findings of this study lays a foundation on how to set priorities in planning oral health promotion activities. Both the educational and policy aspects of health promotion have to be sensitive to these disparities in order to enable disadvantaged rural communities to live healthier lives.

## Conclusion

The findings of this study demonstrated social demographic disparities in relation to oral health related behaviors, while dental pain was associated with low consumption of sugar.

## Competing interests

The authors declare that they have no competing interests.

## Authors' contributions

JRM: participated in conception and design of study, data collection, analysis, interpretation and writing the manuscript. EK: participated in the design of the study, data collection and critical revision of the manuscript. FK: participated in reviewing the proposal, data collection, and revision of the manuscript. IK: critically reviewed the manuscript. AS formulated the research question, participated in data acquisition and critically reviewed the manuscript

## Pre-publication history

The pre-publication history for this paper can be accessed here:


